# Gestation and COVID-19: clinical and microbiological observational study (Gesta-COVID19)

**DOI:** 10.1186/s12884-021-03572-4

**Published:** 2021-01-22

**Authors:** Anna Suy, Itziar Garcia-Ruiz, Melchor Carbonell, Pablo Garcia-Manau, Carlota Rodo, Nerea Maiz, Elena Sulleiro, Andres Anton, Juliana Esperalba, Nuria Fernández-Hidalgo, Marie Antoinette Frick, Fatima Camba, Tomas Pumarola, Elena Carreras, On behalf of the Gesta-COVID19 Collaboration Group, Anna Suy, Itziar García-Ruiz, Melchor Carbonell, Pablo Garcia-Manau, Carlota Rodo, Nerea Maiz, Elena Sulleiro, Andres Anton, Juliana Esperalba, Nuria Fernández-Hidalgo, Marie Antoinette Frick, Fatima Camba, Tomas Pumarola, Elena Carreras, Manel Mendoza, Lidia Illan, Lorena Fernandez-Blanco, Francisco José Barranco, Paula Garcia-Aguilar, Silvia Arevalo, Berta Serrano, Marina Catalan, Helena Tur, Maria de la Calle, Juan Luis Delgado, Leire Rodriguez, Sara Ruiz-Martínez, Francisca Sonia Molina, Vicente Diago, Silvia Mateos

**Affiliations:** 1 Department of Obstetrics, Hospital Universitari Vall d’Hebron, Barcelona, Spain; 2 Department of Obstetrics, Hospital Universitari Vall d’Hebron, Universitat Autònoma de Barcelona, Barcelona, Spain; 3 Maternal-Fetal Medicine Unit, Department of Obstetrics, Vall d’Hebron University Hospital, Ps Vall d’Hebron 119-129, 08035 Barcelona, Spain; 4 Department of Microbiology, Hospital Universitari Vall d’Hebron, Barcelona, Spain; 5 Infectious Diseases Department, Hospital Universitari Vall d’Hebron, Universitat Autònoma de Barcelona, Barcelona, Spain; 6 Red Española de Investigación en Patología Infecciosa (REIPI), Instituto de Salud Carlos III, Madrid, Spain; 7 Department of Pediatric Infectious Diseases and Immunodeficiencies Unit, Hospital Universitari Vall d’Hebron, Barcelona, Spain; 8 Department of Neonatology, Hospital Universitari Vall d’Hebron, Barcelona, Spain

**Keywords:** SARS-CoV-2, COVID-19, Pregnancy, Vertical transmission, Amniotic fluid, Placenta, Cord blood, Breastmilk

## Abstract

**Background:**

The Coronavirus Disease 2019 (COVID-19) is a novel disease which has been having a worldwide affect since December 2019. Evidence regarding the effects of SARS-CoV-2 during pregnancy is conflicting. The presence of SARS-CoV-2 has been demonstrated in biological samples during pregnancy (placenta, umbilical cord or amniotic fluid); however, maternal and fetal effects of the virus are not well known.

**Methods:**

Descriptive, multicentre, longitudinal, observational study in eight tertiary care hospitals throughout Spain, that are referral centres for pregnant women with COVID-19. All pregnant women with positive SARS-CoV-2 real-time reverse transcriptase polymerase chain reaction during their pregnancy or 14 days preconception and newborns born to mothers infected with SARS-CoV-2 will be included. They will continue to be followed up until 4 weeks after delivery. The aim of the study is to investigate both the effect of COVID-19 on the pregnancy, and the effect of the pregnancy status with the evolution of the SARS-CoV-2 disease. Other samples (faeces, urine, serum, amniotic fluid, cord and peripheral blood, placenta and breastmilk) will be collected in order to analyse whether or not there is a risk of vertical transmission and to describe the behaviour of the virus in other fluids. Neonates will be followed until 6 months after delivery to establish the rate of neonatal transmission. We aim to include 150 pregnant women and their babies. Ethics approval will be obtained from all the participating centres.

**Discussion:**

There is little information known about COVID-19 and its unknown effects on pregnancy. This study will collect a large number of samples in pregnant women which will allow us to demonstrate the behaviour of the virus in pregnancy and postpartum in a representative cohort of the Spanish population.

## Background

Severe Acute Respiratory Syndrome Coronavirus 2 (SARS-Cov-2) was first reported in the city of Wuhan, China, in December 2019, with a rapid spread to the rest of the planet within a few weeks. Spain reported its first case of the Coronavirus Disease 2019 (COVID-19) in February, 2020 and as of December 29th 2020, almost 2 million cases have been reported [1].

Evidence regarding the effects of SARS-CoV-2 during pregnancy is conflicting: A study on pregnant women in the United States of America reported that percentages of mild, moderate and severe disease for pregnant women are similar to that of the general population (80, 15 and 5%, respectively) [2] whilst others suggest higher rates of Intensive Care Unit (ICU) admission and oxygen supplementation in pregnant women above 20 weeks’ gestation compared to non-pregnant population with COVID-19 [3]. Many publications regarding the maternal-fetal effects of the virus have raised: a preeclampsia-like syndrome in women affected by COVID-19 has been described [4] and transplacental transmission and possible fetal effects of the virus have been demonstrated [5–7]. The presence of the virus in amniotic fluid and breast milk has also been reported [6, 8]. However, evidence on the effects of the SARS-CoV-2 infection during the first trimester of pregnancy is scarce, with only few publications reporting low rates of miscarriage in the first trimester [9] In our centre we have identified patients with these characteristics, the obstetric and neonatal results of which will be of utmost importance for the correct care and treatment of this disease in pregnant women. Given the little evidence published so far regarding the effects that COVID-19 can have on pregnancy and vice versa, it is essential to gather as much information as possible.

We designed an observational study in 8 tertiary care hospitals in Spain, that are referral centres for pregnant women with COVID-19 during the pandemic. The study intends to investigate both the effect of COVID-19 on pregnancy, and the effect of pregnancy status with the evolution of SARS-CoV-2 disease.

## Methods/design

### Design and setting of the study

This is a descriptive, multicentre, longitudinal, observational study in eight tertiary care hospitals throughout Spain, that are referral centres for pregnant women with COVID-19: Vall d’Hebron University Hospital (HUVH), Barcelona (coordinating centre); La Paz University Hospital (HULP), Madrid; Hospital Virgen de la Arrixaca (HUVA), Murcia; Hospital Universitario Cruces (HUC), Bizkaia; Hospital Clínico Universitario Lozano Blesa (HCULB), Zaragoza; Hospital de Torrejón, Madrid; Hospital Universitario San Cecilio, Granada; and Hospital Universitario La Fe, Valencia.

Given that COVID-19 is an emerging disease and our knowledge of it evolves from day to day, authors will be encouraged to add other variables to the protocol as new clinical evidence comes forward, with the commitment to notify any changes in protocol to the Ethics Committee.

### Objective

To investigate both the effect of COVID-19 on pregnancy and the effect of pregnancy status with the evolution of SARS-CoV-2 disease.

### Outcomes (Table 1)

**Table 1:** Definition of primary and secondary outcomes

**Primary outcome**	
Preeclampsia, preterm delivery, need of hospitalization, ICU admittance	Frequency of each complication / total number of pregnant women
**Secondary outcomes**	
Description of COVID-19 maternal symptoms	Frequency of each symptom / Total number of pregnant women
Maternal mortality	Number of maternal deaths due to COVID-19 or any other complication during the pregnancy / Total number of pregnant women
Rate of fetal morbidity and mortality	Number of miscarriage, stillbirth, fetal malformation, intrauterine growth restriction / Total number of foetuses
Description of behaviour of the virus in biological fluids (urine, faeces, blood cord, placenta and breastmilk)	Number of positive tests / Number of tests performed (per each fluid or tissue)
Neonatal infection at 24, 48 h and 7 days	Number of newborns with positive RT-PCR in pharyngeal aspirate at 24, 48 h and at 7 days / Total number of newborns
Neonatal mortality	Number of neonatal deaths within the first 7 (early) and 28 days of life / Total number of foetuses/newborns
Neonatal morbidity (pneumonia, NICU admission, sepsis,…)	Number of each complication / Total number of neonates

*ICU* Intensive Care Unit, *NICU* Neonatal Intensive Care Unit

Primary outcome:

- Rate of perinatal morbidity in pregnant women with COVID-19 (preterm delivery, preeclampsia, hospitalization during pregnancy, and admission to the ICU)

Secondary outcomes:

- Description of COVID-19 maternal symptoms
- Rate of maternal mortality
- Rate of fetal mortality
- Rate of fetal morbidity (miscarriage, stillbirth, fetal malformation, and intrauterine growth restriction)
- Description of the behaviour of the virus in biological fluids (peripheral blood, urine, faeces, blood cord, placenta, and breastmilk) and serological response
- Rate of neonatal infection at 24, 48 h and 7 days after birth
- Rate of neonatal mortality
- Rate of neonatal morbidity (infection, pneumonia, and admission to the ICU)

### Participants and processes

All consecutive pregnant women with a confirmed COVID-19 diagnosis by real-time reverse transcriptase polymerase chain reaction (RT-PCR) will be included.

Women attended in a labour ward or outpatient clinic with SARS-CoV-2 symptoms (fever, cough, dyspnoea, anosmia, ageusia, diarrhoea, fatigue, myalgias) [10] or those in close contact with a COVID-19 confirmed case, will undergo a SARS-CoV-2 RT-PCR in nasopharyngeal and oropharyngeal smears.

They will be managed according the presence of mild (fever, cough, anosmia, diarrhoea), moderate (tachypnoea >30xmin, hypoxia (saturation < 93% in room air at sea level or PAO2/FiO2 < 300 mmHg) or abnormal chest imaging (> 50% affected lung) or severe symptoms (respiratory failure, shock) [10, 11] Women with mild symptoms will be managed as outpatients and called in daily for a symptoms’ evaluation until a SARS-CoV-2 RT-PCR negativization. Women requiring hospitalization (with moderate or severe symptoms) will be managed according to gestational age and symptoms until discharged from the hospital.

All women with a previous history of COVID-19 and negative RT-PCR SARS-CoV-2 will be followed monthly in the outpatient clinic until delivery and at 4 weeks postpartum.

Delivery of positive SARS-CoV-2 women will be performed according the Spanish Guidelines for COVID-19 pregnancy and delivery [12, 13]. Neonates will not be separated from their mothers but women will be instructed regarding measures to prevent contagion of their sons.

Inclusion and exclusion criteria:

- Inclusion criteria: women with SARS-CoV-2 RT-PCR during pregnancy or 14 days preconception and newborns born to mothers infected with SARS-CoV-2.
- Exclusion criteria: refusal to participate in the study, pregnant women under 18 years of age, and difficulty to understand informed consent.

**Fig. 1:**
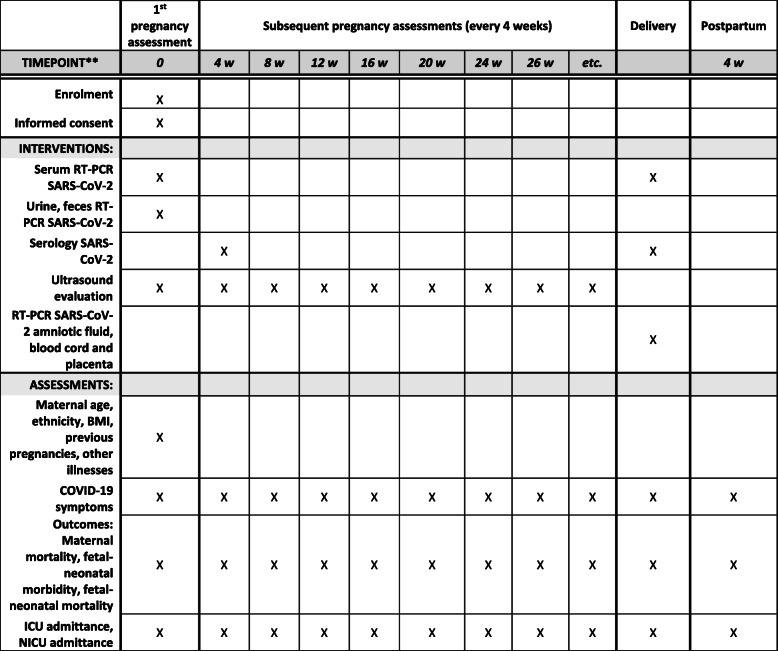
Gesta-COVID19 Study protocol. Pregnant women timeline (SPIRIT figure)

Samples to be collected (Fig. 1):

- Pregnant women: SARS-CoV-2 RT-PCR in nasopharyngeal and oropharyngeal swab at first visit, weekly until SARS-CoV-2 RT-PCR negativization, delivery and postpartum; SARS-CoV-2 RT-PCR in amniotic fluid, urine, faeces, peripheral blood and serum for serologic tests, according to clinical criteria; SARS-CoV-2 RT-PCR in amniotic fluid, placenta, cord blood, and breast milk at delivery and postpartum. Serologic tests within 4–6 weeks after negative SARS-CoV-2 RT-PCR results.
- Newborns born to COVID-19 women: SARS-CoV-2 RT-PCR in nasopharyngeal aspirate, urine, and faeces after delivery. Serologic tests at delivery and after 30 days and 6 months postpartum.
- Newborns born to COVID-19 women requiring admission to the Neonatal Unit: SARS-CoV-2 RT-PCR in nasopharyngeal aspirate and tracheal aspirate (if intubated) after delivery, 24 h, 5 days, and 14 days after birth. SARS-CoV-2 RT-PCR in urine, faeces and serologic tests after delivery. Serologic tests after 30 days and 6 months postpartum.

The detection of SARS-CoV-2 RNA for any sample type will be performed by means of a commercial RT-PCR-based assay Allplex™ 2019-nCoV (Seegene, Korea). The serological study consists in the determination of specific IgG antibodies for pregnant women and specific IgG and IgM antibodies in newborns. These tests will be determined from serum samples using Liaison SARS-CoV-2 S1/S2 IgG (DiaSorin, Italy) and Liaison SARS-CoV-2 S1-RBD IgM. Both of them will be performed on the LIAISON® XL Analyzer (DiaSorin, Italy).

Data will be collected and encoded on an electronic data collection sheet (REDCap®). Each patient will have an identification number for the study. The correlation between the medical record number and the study identification number will be collected in a separate and anonymized database.

### Statistics

A descriptive analysis for the primary and secondary outcomes will be carried out, calculating absolute and relative frequencies. For the maternal characteristics’ description, median and interquartile range will be used for continuous variables and absolute and relative frequencies for categorical variables.

Logistic regression analysis will be used to explore the association of adverse maternal, fetal and neonatal outcomes with each specific drug used to treat COVID-19.

All analysis will be carried out with R software. Statistical significance will be set at *p* < 0.05.

#### Sample size

Given the short evolution of this infection, this study aims to be exploratory and to make a quick characterization of the described outcomes. To this end, it has been estimated that within a period of 4 to 6 months (peak of the epidemic and subsequent months), we will be able to achieve a sample size of 150 pregnancies.

### Ethics

This study was approved by the Ethics Committee of the coordinating centre (PR(AMI)181/2020) and by that of all the participating centres.

Patients will be informed about the study. Given the current situation of the pandemic and public health concerns (in order to reduce the risk of contagion), the Ethics Committee accepted to obtain oral consent from the patient and to record it in the medical history.

## Discussion

The mother-foetus binomial is unique in medicine, morbidity and mortality can therefore affect both. This prospective longitudinal study aims to collect all consecutive cases of COVID-19 in pregnant women in eight referral centres throughout Spain, with the objective of defining whether the pregnancy implies a change in the prognosis of the infection, and vice versa, whether the infection impacts the pregnancy. Furthermore, we intend to assess vertical transmission.

The electronic data collection sheet, accessible via the website from eight of the main reference hospitals in Spain, will allow us to obtain a representative sample of the pregnant Spanish population.

The major strength of this study is that weekly consecutive samples of the naso / oropharyngeal smears will be obtained, allowing to infer virus clearance. Collecting other samples apart from the naso / oropharyngeal smears (peripheral and cord blood, serum, faeces, urine, amniotic fluid, placenta, breast milk and serologic follow-up), will grant more information on the behaviour of the virus in other biological fluids and investigate whether or not there is risk of perinatal transmission.

Due to the limited information on COVID-19 in pregnancy to date, the limitation in the management and treatment of pregnant women, and the possibility of a new outbreak when confinement measures will relax, it is necessary to collect all possible evidence to establish management protocols and working dynamics to efficiently react when it happens. It is the clinicians’ responsibility to be updated on COVID-19 in order to be able to offer the best care to their patients.

## Data Availability

The datasets used and/or analysed during the current study are available from the corresponding author on reasonable request.

## Abbreviations

SARS-CoV-2: Severe acute respiratory syndrome coronavirus 2
COVID-19: Coronavirus Disease 2019
SARS-CoV: Severe Acute Respiratory Syndrome Coronavirus
MERS-CoV: Middle East Respiratory Syndrome Coronavirus
HUVH: Vall d’Hebron University Hospital
HULP: La Paz University Hospital
HUVA: Hospital Virgen de la Arrixaca
HUC: Hospital Universitario Cruces
HCULB: Hospital Clínico Universitario Lozano Blesa
RT-PCR: Real-time reverse transcriptase polymerase chain reaction
EC: Ethics Committee
